# Humanized professional training with Shantala massage workshops: dialogues between theory, technique, and practice

**DOI:** 10.1590/1980-220X-REEUSP-2025-0258en

**Published:** 2026-04-17

**Authors:** Maria das Graças Barreto da Silva, Vitória Helena Cunha Espósito, Conceição Vieira da Silva Ohara

**Affiliations:** 1 Universidade Federal de São Paulo, Escola Paulista de Enfermagem, Departamento de Enfermagem Pediátrica, São Paulo, SP, Brazil.; 2 Pontifícia Universidade Católica de São Paulo, Faculdade de Educação, São Paulo, SP, Brazil.

**Keywords:** Shantala, Human Development, Pediatric Nursing, Qualitative Research, Education

## Abstract

**Objective:**

To understand the experiences lived by professionals and students participating in baby massage workshops: one of the activities of the Baby Massage and Stimulation (BMS) extension program.

**Method:**

A phenomenological hermeneutic trajectory was undertaken with the question: what was your experience like in the baby massage workshop? At the request of the Health Promotion Program, Shantala massage workshops were conducted in the city of São José dos Campos, SP, aiming to implement the BMS program’s educational action, characterized by Baby Massage and Stimulation Therapeutic Groups in the routine of maternal and child care in Basic Health Units.

**Results:**

The meanings given to the experiences lived by workshop participants point to perspectives that help them in their ability to establish human relationships in professional spaces. Participants construct themselves, situate themselves, and create culture, realizing that the bond is also moral, contextual, ethical, and political.

**Final considerations:**

Dialogues between theory, technique, and practice are proposed in BMS program training settings, organizing and integrating new knowledge with existing knowledge, aiming at transformations.

## INTRODUCTION

As a culturally expressive knowledge that has been historically reinterpreted, baby massage originating from India, called Shantala massage (SM)^(1)^, has provided the basis for a body-based approach to work, carried out systematically by the university extension program Baby Massage and Stimulation (BMS)^(2)^. Anchored in knowledge of affective neuropsychomotor development in children, the BMS program consists of two educational actions: the Baby Massage and Stimulation Therapeutic Group (BMSTG), which welcomes parents with their infant babies; and SM workshops, with experiences that introduce health and early childhood education professionals and undergraduate students to SM.

These workshops, in addition to providing consulting services to professionals and institutional services, have an itinerant character. In the academic sphere, as a way to prepare students to work with families, the BMS program is organized into two projects: “Baby Massage and Stimulation Study Group”, an ongoing program; and “SM: multimodal intervention and somatosensory perception”, a theme chosen annually by the monitors for their training^(3)^.

The experiences lived since its origin, in 1996, still as an extension project^(4)^, continued as the “BMS program”, from 2012 onwards, with educational actions in outpatient clinics focused on Primary Care in pediatric and neonatal hospital units, as well as daycare centers, in addition to other academic activities developed with undergraduate courses in the lecture halls of the School of Nursing.

Literature on SM^(5)^ shows that the demand for this type of intervention is gaining followers, and its therapeutic use is becoming increasingly widespread, including in Neonatal Intensive Care Units^(6,7)^. However, these units often still lack humanized approaches that consider health as the ultimate goal of care and that are geared towards the empowerment of families. From this perspective, it is also noteworthy how professionals seek to express their dissatisfaction with what generally occurs in institutions – both health and early childhood education – where babies’ expressions, as well as children’s attitudes, are not used as strategies for caring for them.

Thus, as healthcare and early childhood education professionals, students, and families become aware of the quality of touch in caregiving, the importance of SM-mediated intervention, and its effects on child development (CD), the affective bond will consequently be strengthened^(8,9)^. However, with the expansion and diversification of research focused on these themes, the aim is to achieve recognition of educational action, as well as its appreciation as a soft technology due to its essential benefits within the range of care that comprise parenthood^(10)^.

In response to these desires, SM workshops have sought to make knowledge related to CD accessible and, thus, through SM^(11)^, to highlight to professionals and students the need to review care routines and other procedures using knowledge of children’s age groups through dialogue and reflection.

The Ministry of Health, with the publication of Ordinance 971, approved the Brazilian National Policy on Integrative and Complementary Practices, with a focus on care within the Unified Health System, which outlines the guidelines and responsibilities for implementing its actions and services nationwide^(12)^. In Brazil, there has long been a growing demand from the population for the legitimization of a holistic therapeutic approach, including a demand for Integrative and Complementary Health Practices (ICHP)^(13)^. With the publication of Ordinance 849 in 2017, the Ministry of Health expanded the scope of ICHP and incorporated SM, including the maternal and child population^(14)^. The legal framework represented a means of implementing a strategy to promote access, highlighting a major achievement for public health.

Considering the social responsibility of academia in training health and early childhood education professionals for humanized care^(15)^, we sought to contribute to the improvement of services as participants in public policies oriented towards early childhood, joining efforts so that the law, which specifies the relevance of attention to CD, can be expressed in actions^(16)^.

The dissemination of knowledge produced through the actions developed by the BMS program has been systematically presented to the scientific community, with one of its results being the work “The meanings of experiences lived in the massage and stimulation with babies extension program”^(17)^, from which the excerpt presented here was taken, themed as “Support for humanized professional training: dialogues between theory, technique, and practice”.

This study aimed to consolidate BMS program practices and understand the experiences of those who participated in the SM workshop.

## METHOD

### Study Design

This investigation highlights the phenomenological approach, since academic studies should not be restricted solely and primarily to their technical-scientific aspects. It is considered that nursing and education can benefit from the breadth of care focused on the human dimension as a being existentially situated in the world. In this study, existential and hermeneutic phenomenology was chosen, as this epistemology allows for a qualified perspective on one of the educational actions developed by the BMS program. Phenomenology seeks to understand human beings in their complexity “[...] as beings situated in the world with others, allowing them to be approached from their varied perspectives”^(18)^.

This trajectory is supported by classical authors^(19)^, as well as contemporary thinkers^(18,20,21)^, each with their own phenomenological approach, with their own methodological rigor, characteristic of this way of investigating. From this perspective, it is highlighted how knowledge arises from a certain place, i.e., in the world as focused on here, in a situation^(22)^.

With the introduction of phenomenology into hermeneutics, we observe being in existence, in what is called ontology, i.e., the being conceived as having a common characteristic, inherent to all and each of the beings^(19)^. Thus, by making visible the structure of being in the world, it rests on the understanding and interpretation of how things manifest themselves^(18)^, allowing one to understand the meanings of the experiences lived in the baby massage workshops.

This study comprehensively illuminated the work developed in the BMS program, allowing participants to grasp its meaning. Systematized within the workshops, the educational action brought the possibility of constructing its own methodological trajectory^(17)^ by emphasizing praxis, i.e., the conscious experience of individuals in their life-world, undertaken with students and professionals.

### Location, Population, and Inclusion Criteria

This methodological trajectory refers to the elements responsible for the text’s pertinence and relevance in relation to the context in which it occurs, i.e., its adequacy to the socio-communicative situation. It encompasses how participants’ descriptions were accessed, including, as a research area, workshop V. This term refers to the thematic area, i.e., the set of delimited experiences that are being investigated through the phenomenological methodology^(23)^.

At the request of the coordination of the Health Promotion Program/Center for Integrative and Complementary Practices/Department of Health Policies of the Municipal Health Department, five SM workshops were held in the city of São José dos Campos, SP. These workshops, aimed at healthcare professionals and nursing students, sought to train multipliers to work with SM integrated into the work they already develop with pregnant women and in the childcare segment. In this way, the BMSTG implementation in the routine of maternal and child healthcare by the primary and secondary care network was envisioned, in Basic Health Units (BHUs) and in a Lactation Center Unit, where they were already working.

### Data Collection

Phenomenological descriptions were collected by the researcher at the end of each event in workshops I, II, III, IV, and V, at which time participants answered the following question: how was your experience in the SM workshop?

Since workshop V was the last one offered, randomly selected, we began by reading the descriptions of its participants, choosing it as the research area.

Since qualitative research does not require a specific number of participants, the study could be concluded when the meanings already found expressed satisfactory content for understanding its essential structures^(24)^. Initial readings of the descriptions provided by participants in other workshops were also conducted, but no relevant meanings were found, i.e., none that added elements to understanding the phenomenon. Therefore, it can be considered that an ideal qualitative sample is one that reflects, in quantity and intensity, the multiple dimensions of a given phenomenon, seeking the quality of actions and interactions throughout the process^(25)^.

Inclusion criteria for the descriptions of the 12 professionals and one student – participants in the workshop – considered their participation in both modules of workshop V. Thus, the trajectory was carefully followed, focusing on their descriptions and their perspectives. To this end, without judgment, opinions, assumptions, and interpretations were suspended, focusing instead on their lived experiences, i.e., on the relevant meanings that marked the phenomenon “[...] in response to the research question”^(17)^.

### Data Analysis and Processing

From this perspective, understanding was achieved through the meaning that emerged as a result of phenomenological reduction, being understood as a set of significant assertions called units of meaning (UMs), which point to the researcher’s awareness of the phenomenon^(23)^. Thus, with the support of lexicons^(26)^, care was taken to ensure that the UMs reflected certain dimensions of the context. After numerous readings of the different descriptions of the 13 participants in workshop V – identified as D (description) I to XIII (with Roman numerals referring to the workshop participants) –, the first phenomenological reduction was reached. From this, 1 to 59 UMs (presented by cardinal numbers) were derived in response to the research question. After they were organized, they generated syntheses, which are open to interpretation.

Proceeding with a second reduction, 15 themes were visualized from the phenomenon’s essential elements, expressed as follows: theory, technique, and practice; learning; didactics; methodological strategy; interdisciplinary perspective; purpose; expectation; discovery; awakening; lack; congratulation; satisfaction; care; assessment; and gratitude. Due to their interrelationships, by making convergences explicit, an attempt was made to create a texture from these themes, which resulted in the reorganization of large categories that, subsequently, with the realization of a third reduction, point to the themes, expressed as follows: (1) Support for humanized professional training: dialogues between theory, technique, and practice; (2) Contribution to constructing a mother/father-child bond and caregiving professionals; and (3) Time lived in the BMS workshop experience: unveiling meanings. However, for this work, it is presented expressed in the first theme presented below.

### Ethical Aspects

The design of this study was approved by the *Universidade Federal de São Paulo* Research Ethics Committee, under Opinion 1,424,084, and complied with the ethical precepts of Resolution 466 of December 12, 2012 of the Brazilian National Health Council^(27)^.

## RESULTS AND DISCUSSION

Considering the SM workshop as a space in search of meaning, what emerged was expressed in the theme “Support for humanized professional training: dialogues between theory, technique, and practice”, based on the themes “theory, technique, and practice”, “learning”, “didactics”, “methodological strategy” and “interdisciplinary perspective”.

In this methodological journey, when facing the challenge of writing, which is pedagogically oriented towards lived experience, the following idea was shared: the act of writing reflectively about the practice of living allows us to become engaged in a more reflective praxis^(20)^. It is therefore considered that it is through writing and reading that a conversational relationship can be sustained; thus, this is related to language and writing quality. In other words, it is linked to the researcher’s ability to give meaning to the topic under study (in theory). Thus, language is the only means by which we can convert the pedagogical experience into a symbolic form that, through its discursive nature, allows us to create a conversational relationship^(20)^.

However, the knowledge of praxis relating to the sciences of the spirit, in which ethics is the driving and guiding element of our doing^(28)^, when based on norms and rules — i.e., on morality —, begin to guide the different forms of conduct, both of those who conduct and those who perform and receive the practical action. Thus:

The praxeological knowledge of phronesis, guided by the excellence of actions that find the best “means” to achieve a just and ethical end, focuses on humankind and its experiences, without being bound by the requirement of objectivity or proof of what it is, considering it as a being of possibilities, a being situated in time and space, contextualized, historical, living in a world of uncertainties^(28)^.

However, within the ethical knowledge of practice (phronesis) lies the perception of how the concept of care is differentiated in the workshop setting. There, the dynamic is developed between professionals and professors, using dolls for learning the SM technique in practice. It is observed that the instructions offered serve as a guide, because, as they are not definitive, when in SM sessions with families, they can be adapted or modified according to the context and circumstances expressed by participants. Therefore, attention is drawn to participants observing parents’ reactions and expressions in relation to touches so that their signs of pleasure and displeasure are respected. Similarly, babies’ reactions are observed, considering their preferences, as this favors the improvement of the practical action, as well as care students’/professionals’ understanding, assisting them in making better choices.

Furthermore, considering the work developed within the BMS program, the importance of each team member recognizing themselves as a holder and producer of significant knowledge in the development of care actions is highlighted, establishing exchanges through systematic dialogues, in a process where sharing experiences is paramount^(29)^.

This atmosphere, conducive to the exchange of experiences and concepts, allows for a reinterpretation of the meanings given to lived experiences, making it possible to respect workshop participants’ spontaneity and enhancing communication between civic and professional actions.

Since the participants surveyed are professionals and students with a background of knowledge, when faced with new information that makes sense - here taken in the sense of “awareness of”^(22)^ - they make an effort to assimilate it. Thus, when faced with a problem, they indicate the need to overcome it, as stated: “*Modern human beings lack touch*” (DVII.UM.5); “*Especially with affection*” (DVII.UM.5.1); “*This promotes a culture of peace*” (DVII.UM.5.2).

From the previous description, it is understood that the individuals demonstrates an awareness of how much people today lack contact with others, especially with care, and shows confidence in SM as a strategy to promote human interactions and as a driving force for the development of a culture of peace.

In this regard, “[...] education is highlighted as a form of awareness, mobilization and prevention at all social levels, and children as subjects who can bring peace from the realm of imagination to reality, when cared for with love, which can favor subsequent developments”^(30)^.

However, there is participant identification, as mentioned in the description, and consideration of how the SM theme is conveyed by the workshop, indicating recognition of its benefits for the community: “*I found the proposed theme extremely interesting* [...] *and very useful*” (DV.UM.1).

Considering its theoretical dimension, in the lexicon the.o.ry^(26)^ (from the Greek, *theoría*), feminine noun, refers to the basic and elementary principles of an art or science. Here, SM, combined with some principles, qualifies the dialogue between theory, technique, and practice of the pedagogical actions experienced in the BMS program.

The.o.ry can also be understood as a system [...] that deals with principles, considering sys.te.m^(26)^ (from the Greek, *sýstema*) as method, mode, form or plan and prin.ci.ple^(26)^ (from the Latin, *principium*), here seen as the foundation, basis of SM.

These theoretical principles are also intertwined with the tech.ni.que^(26)^ (feminine noun in Portuguese), i.e., with the special way or skill of performing or even teaching, SM can be seen in the description of another workshop participant: “*I really liked the technique*” (DVI.UM.1).

These are theoretical and technical principles that, when applied to SM, also characterize the teaching methods employed, i.e., the practical action as used by the BMS program, which gradually enables the achievement of the desired effects. For its understanding, it is suggested that professionals put themselves in the place of mothers/babies – a teaching strategy used when presenting the technique –, i.e., consider teaching the SM stages in a way that it is performed together, placing everyone as subjects of the educational action.

From the participating professionals’ perspective, the workshop experience brought discoveries: “[...] *a complementary therapy technique that I did not know in depth*” (DV.UM.2). Participants unveiled SM as a methodological strategy capable of bringing mother and child closer, contributing to optimization of care: “*A tool to improve the mother-child bond and thus improve areas of care*” (DIX.UM.2); “*This simple technique that can greatly help in contact with mother and child*” (DX.UM.2).

Looking at its practical dimension (from the Greek, *praktiké*, feminine noun), the application of the rules or principles of an art or science is located in the lexicon^(26)^. From that same perspective, Leboyer, when reporting to SM, wrote the following poem that was freely translated into English:

Baby massage is an art

as ancient as it is profound.

Simple yet difficult.

Difficult because it is simple.

Like everything that is profound^(1)^

The lexicon^(26)^ also finds prac.ti.ce as a skill [...] acquired through prolonged practice – in this case, shared with workshop participants. Thus, “Practical knowledge, unlike theoretical knowledge, cannot be generalized and can be used as a guideline and orientation^(22)^. This skill is achieved in a unique way by doing, doing, doing...”

In his description, a workshop participant suggests: “*And the practical part was very good too*” (DV.UM.4). The use of puppets for learning was considered appropriate, which leads to the phenomenon of education being illuminated in the workshop’s space-time.

Following the descriptions, it is noted that a participant highlights the practice of SM as “an act of love”, an appropriate occasion for exchanging information with the baby through the senses, as well as for observing and perceiving the baby: “*Massage is an act of love, a moment to share experiences with the baby, to understand and respect them. This interaction only brings benefits and needs to be valued and promoted*” (DVII.UM.4). In this way, the workshop participant demonstrates an awareness of reciprocity and the advantages that this practice entails by emphasizing that it should be encouraged, shared, and expanded.

In this context, it can be said that: “Practice, in addition to requiring prior knowledge that favors the theoretical-technical-practical character in dialogue, demands other knowledge that [...] serves as an (ethical) guide”^(22)^. These are areas of knowledge whose importance was identified in the descriptions as follows: “[...] *the information regarding sensory and psychomotor development that underpins the fundamentals of massage is very rich*” (DI.UM.3); *“Furthermore, it clearly shows the benefits and strengthening of the emotional bond with the baby*” (DI.UM.4).

The professional indicates, in another description, that although he expected to learn only the SM stages, he found that the knowledge gained was extensive, based on the theory provided about CD: “[...] *it surprised me; I was only expecting to learn the practical aspects of massage*” (DI.UM.2). This knowledge, coupled with an understanding of the advantages and effectiveness of the intervention for fostering an affectionate bond between mother and baby, allows for the qualitative advancement of knowledge in a procedural and dynamic way. Although SM is a systematized touch on the skin, not isolated from the other senses, it can be considered a multimodal intervention, as it promotes integration between the senses and motor skills^(17)^.

In keeping with the principles of Freirean socio-political pedagogy^(29)^, it can be seen that the work has been developed in a committed manner through a dialogical relationship that focuses on egalitarian, horizontal education. This shows that theoretical, technical, and practical knowledge, when integrated, has equal relevance.

Thus, by reflecting on what often occurs in music, resorting to a metaphorical dimension or even an imaginative variation, one can glimpse when voices or musical instruments alternate or even establish a dialogue with each other. As in jazz, it is possible to refer to the intricate relationship between the theory, technique, and practice of SM, which also allows us to unveil a dialogue: where the theory carries the ancient knowledge of oriental massage, combined with the principles listed by the BMS program for its implementation. The technique, in turn, is characterized by the systematic oral transmission of the SM stages, from the East, from generation to generation, allowing it to reach the West and continue to be practiced and taught to this day. Practice is expressed in pedagogical action through SM, characterized by its effectiveness and supported by the CD theory, based on the continued application of the technique in the BMS program, adapted and shared with families, students, and professionals.

However, the methodological choice and the selection of theoretical frameworks brought to the focus of the theme, whether observational, learning-based or discovery-based, along with motivational factors, indicate the value placed on the subjects involved in the SM actions offered by the workshops.

Therefore, considering SM’s educational action in this way requires a concern for education within the broader context of valuing human relationships, as highlighted in another description: “[...] *a way to value the figure of the mother*” (DIX.UM.3); and “*Improve the bond with the healthcare professional*” (DIX. UM.4).

As a complementary element, another professional is also observed making reference to SM, not only to make it available to parents with babies, but also as a resource to observe and care for them attentively: “*It will be another tool to* [observe] *and offer to patients*” (DVI.UM.4).

In other words, this means that the understanding that one always knows something refers to the possibility of knowing from lived experience and, therefore, being able to use this knowledge as a starting point to continue, in the process^(29)^, learning and, in this way, maintaining the openness of horizons, which makes it possible to identify what would be necessary for knowledge to advance^(22)^.

This perspective requires professors to study, contextualize, reflect, write, engage in dialogue, and investigate, as their role in the pedagogical environment of extension programs demands the pursuit of knowledge of diverse natures, with defined objectives, through strategies that lead to the construction of original knowledge^(11,17)^.

However, the workshop participant’s admiration for the fundamentals of SM is evident, as he reveals how significant the experience was in terms of learning and admits that it would be good to have access to more knowledge: “[...] *the theoretical part* [...] *was wonderful and I would love to know more*” (DV. UM.3). Finally, it is worth mentioning the description “*The speaker had excellent teaching skills*” (DVIII.UM.1), which provides consideration regarding professors’ work with reference to the pedagogical strategy used.

In this respect, knowledge is seen as a product of action and reflection in the face of a problem (cognitive conflict)^(29)^. This can be supported by workshop participants’ understanding of the complexity of the knowledge that underpins this educational action through SM: “*The topic covers many things*” (DIV.UM.2); “*A knowledge-rich workshop*” (DXI.UM.1).

In this context, the SM workshop reveals itself as a learning tool with diverse purposes: to make professionals and students understand that the dominant paradigm of medical sciences, centered on pathologies, does not respond to some of their desires regarding care, and above all, what the health needs of families are. It also emphasizes the importance of knowledge from other areas of expertise so that participants awaken to the human dimension from an interdisciplinary, ethical, and existential perspective. The workshop presents how care interventions have been conceived and planned, beyond clinical therapy, with actions guided by CD knowledge, mediated by SM and situated within the perspective of the baby’s competencies, who is considered the leading figure of the action. In this way, their expressions and capacities for early interaction and partnership are valued, in order to motivate the search for discovery in young children.

Understanding what can happen to families through the establishment of a daily SM ritual helps reinforce positive attitudes among professionals, sparking interest in facing the challenge of building work in this direction. This is demonstrated by the following testimony, in which the professional states that the workshop sparked her attention, assuring that she (and the team) will set up BMSTG at the BHU where she works: “*Great course, very interesting*” (DXIII.UM.1): “*We will definitely do it at the BHU*” (DXIII.UM.2).

The aim is to fulfill the social function of academia by raising awareness among students and professionals working in daycare centers, early childhood education schools, hospital units, and primary health care clinics. These settings require the development of proposals based on a commitment to interdisciplinarity as a means for professionals to form teams.

In the context of the BMS program, it is believed that, by making it possible to articulate research as an educational action, within the scope of teaching and extension, combining empirical and scientific knowledge on SM guided by CD, there is the possibility of contributing to the training of early childhood professionals, i.e., training directed towards human development^(22)^, focusing on optimization of parenting^(10)^.

Considering affection as a fundamental need of all human beings, indispensable for the balanced construction of personality, literacy in terms of affection and tenderness can be highlighted as a priority objective and a key aspect of the entire educational process. Beyond its influence on the vital process and maturation of individuals, it has an undeniable relationship with coexistence^(30)^.

Hence, when considering the experiences in baby massage workshops, this demonstrates how such educational action can constitute a powerful auxiliary resource for professionals, both in developing their ability to establish intelligent relationships between data and information obtained through experience, and in the realm of constructed knowledge.

### Reflections on the Unveiled Experience

The methodological trajectory of hermeneutic phenomenological research made it possible to illuminate the themes from their varied perspectives, seeking the construction of intersignifications, as well as allowing an understanding of the proposed approach in light of other ways of conceiving scientific studies. A whole was evidenced in which meanings could circulate according to the senses attributed to each one, unveiling the phenomenon of humanized professional training with SM workshops: dialogues between theory, technique, and practice.

## CONCLUSION

The meanings given to the workshops point to perspectives that assist healthcare professionals, both in developing the ability to establish human relationships in professional spaces and in the possibility of opening up to the sharing of lived experiences, added to others that can be built in partnerships, situating the focus of this trajectory in the sociopolitical dimension of affective bonds, which are still poorly understood today.

In the BMS program training settings, in addition to seeking to awaken undergraduate students and professionals – workshop participants – to a more humane care practice, the aim is to develop actions that organize and integrate new knowledge with existing knowledge, aiming at transformations. From this emerges the possibility that they experience their own actions as subjects, because, by situating themselves and interacting, they construct themselves and create culture. They realize that the bond is also moral, contextual/ethical and even political.

## Data Availability

The entire dataset supporting the results of this study is available upon request to the corresponding author.
