# Supplementary material for: Humanized professional training with Shantala massage workshops: dialogues between theory, technique, and practice
Source: Rev Esc Enferm USP. 2026 Apr 17;60:e20250258. doi: 10.1590/1980-220X-REEUSP-2025-0258en (PMC13078790; doi:10.1590/1980-220X-REEUSP-2025-0258en)
Supplement: Translation

# Translation: Formação profissional humanizada com oficinas de massagem Shantala: diálogos entre teoria, técnica e prática

**DOI:** 10.1590/1980-220X-REEUSP-2025-0258pt

**Authors:** Maria das Graças Barreto da Silva, Vitória Helena Cunha Espósito, Conceição Vieira da Silva Ohara

**Affiliations:** 1 Universidade Federal de São Paulo, Escola Paulista de Enfermagem, Departamento de Enfermagem Pediátrica, São Paulo, SP, Brasil.; 2 Pontifícia Universidade Católica de São Paulo, Faculdade de Educação, São Paulo, SP, Brasil.

**Keywords:** Shantala, Desenvolvimento Humano, Enfermagem Pediátrica, Pesquisa Qualitativa, Educação

## Abstract

**Objetivo:**

Compreender os sentidos vividos pelos profissionais e estudantes participantes das oficinas de massagem com bebês: uma das ações do programa de extensão Massagem e Estimulação com Bebês (MEB).

**Método:**

Trajetória fenomenológica hermenêutica com a indagação: como foi sua experiência na oficina de massagem com bebês? Por solicitação do Programa de Promoção da Saúde, ministraram-se oficinas de massagem Shantala na cidade de São José dos Campos, SP, visando à implantação da ação educativa do programa MEB, caracterizada pelos Grupos Terapêuticos de Massagem e Estimulação com Bebês na rotina de cuidados materno-infantil das Unidades Básicas de Saúde.

**Resultados:**

Os sentidos dados às experiências vividas pelos participantes das oficinas apontam perspectivas que os auxiliam na capacidade de estabelecer relações humanas nos espaços profissionais. Os participantes constroem a si mesmos, situam-se e fazem cultura, percebendo que o vínculo é também moral, contextual, ético e político.

**Considerações finais:**

Nos cenários de formação do programa MEB, propõem-se diálogos entre teoria, técnica e prática, ao organizar e integrar novos conhecimentos aos já existentes, visando transformações.

## INTRODUÇÃO

Como um saber culturalmente expressivo, que veio sendo ressignificado historicamente, a massagem com bebês oriunda da Índia, denominada massagem Shantala (MS)^(1)^, propiciou a construção de um trabalho com abordagem corporal, realizado de maneira sistematizada, pelo programa de extensão universitária Massagem e Estimulação com Bebês (MEB)^(2)^. Ancorado nos conhecimentos do desenvolvimento neuropsicomotor afetivo infantil, o programa MEB constitui-se por meio de duas ações educativas: o Grupo Terapêutico de Massagem e Estimulação com Bebês (GTMEB), que acolhe mães/pais com seus bebês lactentes; e as oficinas de MS, com vivências que introduzem profissionais e graduandos das áreas da saúde e educação infantil na temática da MS.

Essas oficinas, além de realizar assessorias a profissionais e serviços institucionais, têm um caráter itinerante. No âmbito acadêmico, como forma de preparo dos estudantes para atuarem junto às famílias, o programa MEB está organizado em dois projetos: “Grupo de Estudo Massagem e Estimulação com Bebês”, de ação contínua; e “MS: intervenção multimodal e a somatosensorialidade”, temática elencada pelas monitoras anualmente para sua trajetória^(3)^.

As experiências vividas desde sua origem, em 1996, ainda como projeto de extensão^(4)^, tiveram continuidade como “programa MEB”, a partir de 2012, com ações educativas em ambulatórios voltados à Atenção Primária em unidades hospitalares pediátricas e neonatais, assim como creches, além de outras atividades acadêmicas desenvolvidas com os cursos de graduação nos anfiteatros da Escola de Enfermagem.

A literatura sobre a MS^(5)^ demonstra que a procura por esse tipo de intervenção está ganhando adeptos, e cada vez mais se difunde seu uso terapêutico, inclusive nas Unidades de Terapia Intensiva Neonatal^(6,7)^. Porém, essas unidades, com frequência, ainda carecem de abordagens humanizadas que considerem a saúde como o fim último dos cuidados e que sejam voltadas ao protagonismo das famílias. Nessa perspectiva, destaca-se também como os profissionais procuram expor suas insatisfações com o que geralmente ocorre nas instituições – de saúde e educação infantil –, onde as expressões dos bebês, assim como as atitudes das crianças, não são utilizadas como estratégias para cuidá-los.

Assim, à medida que profissionais das áreas da saúde e da educação infantil, estudantes e famílias se tornarem conscientes da qualidade do toque nos cuidados, da importância da intervenção mediada pela MS e de seus efeitos sobre o desenvolvimento infantil (DI), consequentemente o vínculo afetivo será fortalecido^(8,9)^. Contudo, com a ampliação e diversificação de pesquisas voltadas a essas temáticas, almeja-se que haja reconhecimento da ação educativa, bem como valorização da mesma como tecnologia leve pelos seus benefícios, essenciais ao rol de cuidados que integram a parentalidade^(10)^.

Em resposta a esses anseios, tem-se buscado, com as oficinas de MS, tornar acessíveis os conhecimentos voltados ao DI e, assim, por meio da MS^(11)^, evidenciar aos profissionais e estudantes a necessidade de revisão das rotinas de cuidados e demais procedimentos utilizando os conhecimentos das faixas etárias infantis por meio de diálogos e reflexões.

O Ministério da Saúde, com a publicação da Portaria nº 971, aprovou a Política Nacional de Práticas Integrativas e Complementares, com abordagem de cuidado no Sistema Único de Saúde, que traça as diretrizes e responsabilidades para a implantação das suas ações e serviços em território nacional^(12)^. Destaca-se, no Brasil, a existência, há tempos, de uma crescente busca da população pela legitimação da abordagem terapêutica holística, incluindo uma demanda pelas Práticas Integrativas e Complementares em Saúde (PICS)^(13)^. Com a publicação da Portaria nº 849, em 2017, o Ministério da Saúde ampliou as PICS e incorporou a MS, contemplando a população materno infantil^(14)^. O marco legal representou um meio de concretizar uma estratégia de promoção do acesso, evidenciando uma grande conquista para a saúde pública.

Considerando a responsabilidade social da academia na formação dos profissionais de saúde e de educação infantil para o cuidar humanizado^(15)^, buscou-se contribuir para a melhoria dos serviços como partícipes de políticas públicas orientadas à primeira infância, unindo esforços para que a lei, que especifica a relevância da atenção ao DI, possa ser expressa em ações^(16)^.

A difusão dos conhecimentos produzidos por meio das ações desenvolvidas pelo programa MEB vem sendo sistematicamente apresentada à comunidade científica, constituindo como um de seus resultados o trabalho “Os sentidos das experiências vividas no programa de extensão massagem e estimulação com bebês”^(17)^, do qual foi retirado o recorte ora apresentado, tematizado como “Apoio à formação profissional humanizada: diálogos entre teoria, técnica e prática”.

A motivação em realizar este estudo, ao visar consolidar os fazeres do programa MEB, possibilitou compreender os sentidos das experiências vividas na oficina de MS por aqueles que dela participaram.

## MÉTODO

### Desenho do Estudo

Destaca-se, para esta investigação, a orientação fenomenológica, uma vez que os estudos acadêmicos não devem se restringir apenas e prioritariamente aos seus aspectos técnico-científicos, pois considera-se que a enfermagem, assim como a educação, possa se beneficiar com a amplitude do cuidado voltado à dimensão humana enquanto ser situado existencialmente no mundo. Neste estudo, optou-se, em particular, pela fenomenologia existencial e hermenêutica, por tal epistemologia permitir um olhar qualificado sobre uma das ações educativas desenvolvidas pelo programa MEB. A fenomenologia procura compreender os seres humanos em sua complexidade “[...] como seres situados no mundo com os outros ao admitir abordá-los em suas variadas perspectivas”^(18)^.

Essa trajetória se encontra amparada em autores clássicos^(19)^, assim como em pensadores contemporâneos^(18,20,21)^, cada um com o seu fazer fenomenológico, com um rigor metodológico próprio, característico dessa maneira de investigar. Nessa perspectiva, ressalta-se como o conhecimento se dá a partir de um certo lugar, isto é, no mundo como aqui enfocado, em situação^(22)^.

Observa-se, com a introdução da fenomenologia na hermenêutica, o ser na existência, na chamada ontologia, isto é, o ser concebido como tendo uma característica comum, inerente a todos e a cada um dos seres^(19)^. Desse modo, ao tornar visível a estrutura do ser no mundo, ela repousa na compreensão e interpretação de como as coisas se manifestam^(18)^, permitindo compreender os sentidos das experiências vividas nas oficinas de massagem com bebês.

Ao se iluminar compreensivamente, este estudo tornou visível o trabalho desenvolvido no programa MEB, permitindo apreender os sentidos de sua construção para aqueles que dele participam. Sistematizada nos espaços das oficinas, a ação educativa trouxe a possibilidade de construção de uma trajetória metodológica própria^(17)^ ao enfatizar a práxis, isto é, a experiência consciente do sujeito em seu mundo-vida, percorrida com estudantes e profissionais.

### Local, População e Critérios de Inclusão

Essa trajetória metodológica se refere aos elementos responsáveis pela pertinência e relevância do texto quanto ao contexto em que ocorre, ou seja, à sua adequação à situação sociocomunicativa. Abarca como foram acessadas as descrições dos participantes, incluindo, como região de inquérito, a oficina V. Esse termo se refere à área temática, ou seja, ao conjunto de experiências delimitadas que estão sendo investigadas por meio da metodologia fenomenológica^(23)^.

Por solicitação da coordenação do Programa de Promoção da Saúde/Núcleo de Práticas Integrativas e Complementares/Departamento de Políticas de Saúde da Secretaria Municipal de Saúde, ministraram-se cinco oficinas de MS na cidade de São José dos Campos, SP. Essas oficinas, destinadas a profissionais de saúde e graduandos de enfermagem, objetivaram formar multiplicadores para operarem com a MS integrada aos trabalhos por eles já desenvolvidos com as gestantes e no segmento com a puericultura. Dessa forma, vislumbrou-se a implantação do GTMEB na rotina de cuidados em saúde materno-infantil pela rede de atenção básica e secundária, nas Unidades Básicas de Saúde (UBSs) e na Unidade Casulo do Centro de Lactação, onde já atuavam.

### Coleta de Dados

As descrições fenomenológicas foram coletadas pela pesquisadora, ao final de cada evento, das oficinas I, II, III, IV e V, momento no qual os participantes respondiam à seguinte indagação: como foi sua experiência na oficina de MS?

Como a oficina V foi a última a ser ofertada, de forma aleatória, iniciamos a leitura das descrições de seus participantes, elegendo-a como a região de inquérito da pesquisa.

Como a pesquisa qualitativa não requer um número determinado de participantes, o estudo pôde ser encerrado quando os significados já encontrados expressaram conteúdos satisfatórios para a compreensão das suas estruturas essenciais^(24)^. Procedeu-se ainda às leituras iniciais das descrições dos participantes das demais oficinas, não tendo sido encontrados significados relevantes, isto é, que acrescentassem elementos à compreensão do fenômeno. Pode-se, assim, considerar que uma amostra qualitativa ideal é a que reflete, em quantidade e intensidade, as múltiplas dimensões de determinado fenômeno, buscando a qualidade das ações e das interações em todo o decorrer do processo^(25)^ *.*

O critério de inclusão das descrições dos 12 profissionais e de uma estudante – participantes da oficina – contemplava suas participações nos dois módulos da oficina V. Desse modo, trilhou-se a trajetória atenta às suas descrições, com os olhos pautados em seus olhares. Para isso, procurou-se, sem julgamentos, manter em suspensão as opiniões, pressupostos e interpretações, com foco nas experiências por eles vividas, isto é, nos significados relevantes que assinalavam o fenômeno “[...] em resposta à indagação da pesquisa”^(17)^.

### Análise e Tratamento dos Dados

Nessa perspectiva, a compreensão se deu por meio do sentido que emergiu como resultado da redução fenomenológica, sendo admitida como um conjunto de asserções significativas que se denominam unidades de significado (USs), as quais apontam para a consciência que o pesquisador tem do fenômeno^(23)^. Seguiu-se, assim, com o apoio do léxico^(26)^, com o cuidado que as USs refletissem certas dimensões do contexto. Após inúmeras leituras das diferentes descrições dos 13 participantes da oficina V – identificadas como D (descrição) I a XIII (com algarismos romanos referindo-se aos participantes da oficina) –, chegou-se à primeira redução fenomenológica. Desta, decorreram de 1 a 59 USs, (apresentadas por números cardinais) em resposta à pergunta da pesquisa. Organizadas, elas geraram sínteses, que estão abertas à interpretação.

Procedendo a uma segunda redução, visualizaram-se 15 temáticas a partir dos elementos essenciais do fenômeno, assim expressas: teoria, técnica e prática; aprendizagem; didática; estratégia metodológica; perspectiva interdisciplinar; propósito; expectativa; descoberta; despertar; falta; congratulação; satisfação; cuidado; avaliação; e agradecimento*.* Em virtude de seus imbricamentos, ao explicitar as convergências, simultaneamente, procurou-se perfazer uma textura a partir dessas temáticas, o que redundou na reorganização de grandes categorias que, na sequência, com a realização de uma terceira redução, apontam para os temas, assim expressos: (1) Apoio à formação profissional humanizada: diálogos entre teoria, técnica e prática; (2) Contribuição à construção do vínculo mãe/pai-filho e profissionais cuidadores; e (3) Tempo vivido na experiência da oficina do MEB: desvelando sentidos. Porém, para este trabalho, apresenta-se o primeiro tema expresso na sequência.

### Aspectos Éticos

O projeto deste estudo foi aprovado pelo Comitê de Ética em Pesquisa da Universidade Federal de São Paulo, sob o Parecer n° 1.424.084, e obedeceu aos preceitos éticos da Resolução nº 466, de 12 de dezembro de 2012, do Conselho Nacional de Saúde^(27)^.

## RESULTADOS E DISCUSSÃO

Contemplando a oficina de MS como um espaço em busca de sentido, emergiu aquilo que fora vivido expresso na temática “Apoio à formação profissional humanizada: diálogos entre teoria, técnica e prática”, a partir dos temas “teoria-técnica-prática”, “aprendizagem”, “didática”, “estratégia metodológica” e “perspectiva interdisciplinar”.

Nessa trajetória metodológica, ao enfrentar o desafio da escrita, que se orienta pedagogicamente à experiência vivida, compartilhou-se a seguinte ideia: o ato de escrever reflexivamente sobre a prática de viver possibilita que nos tornemos engajados em uma práxis mais reflexiva^(20)^. Considera-se, portanto, que é por meio da escrita e da leitura que se pode sustentar uma relação conversacional; logo, isso encontra-se relacionado à qualidade da linguagem e da escrita. Dito de outra forma: encontra-se vinculado à capacidade da pesquisadora de dar sentido ao tema em estudo (em tese). Desse modo, a linguagem é o único meio pelo qual podemos converter a experiência pedagógica em uma forma simbólica que, através de sua natureza discursiva, possamos criar uma relação conversacional^(20)^.

Contudo, os saberes da práxis referentes às ciências do espírito, nos quais a ética é o elemento impulsionador e guia de nosso fazer^(28)^, ao se apoiarem em normas e regras — ou seja, na moral —, passam a orientar as diferentes formas de conduta, tanto de quem conduz quanto de quem realiza e recebe a ação prática. Desse modo:

O saber praxiológico da phronesis, enquanto pautado pela excelência das ações que conseguem encontrar os melhores “meios” para atingir um fim justo, ético, tem como objetivo o homem e suas vivências, sem se pautar pela exigência da objetividade e de comprovação daquilo que ele é, considerando-o como um ser de possibilidades, um ser situado no tempo e no espaço, contextualizado, histórico, vivendo em um mundo de incertezas^(28)^.

Entretanto, encontra-se no saber ético da prática (phronesis) a percepção de como o conceito de cuidado no espaço da oficina é diferenciado. Ali a dinâmica é desenvolvida entre os profissionais e a docente, fazendo uso de bonecas para a aprendizagem prática da técnica da MS. Observa-se que as instruções oferecidas servem como guia, pois, não sendo determinantes, quando em sessão de MS com as famílias, podem sofrer adequações ou modificações, conforme o contexto e as circunstâncias manifestadas pelos participantes. Com isso, chama-se a atenção para que os participantes observem as reações e expressões da mãe/pai em relação aos toques, para que sejam respeitados seus sinais de prazer e desprazer. Da mesma forma, observam-se as dos bebês, considerando suas preferências, pois isso favorece o aperfeiçoamento da ação prática, assim como a do ser estudante/profissional do cuidado, auxiliando-o nas melhores escolhas.

Ademais, diante dos trabalhos desenvolvidos no programa MEB, destaca-se a importância de cada integrante da equipe se reconhecer no desenvolvimento das ações de cuidado como detentor e produtor de saberes significativos, estabelecendo trocas por meio de diálogos sistemáticos, em um processo no qual o intercâmbio de experiências é primordial^(29)^.

Essa atmosfera favorecedora de trocas de experiências e conceitos permite ressignificações dos sentidos dados ao vivido, tornando viável o respeito pela espontaneidade dos participantes da oficina e realçando a comunicação entre ações cidadãs e profissionais.

Como os participantes pesquisados são profissionais e estudantes com bagagem de conhecimentos, diante de novas informações que apresentem sentido - aqui tomado na acepção de “consciência de”^(22)^, realizam um esforço para assimilá-las. Assim, diante de um problema, eles indicam a necessidade de superá-lo, como exposto: “*Falta ao ser humano moderno o toque”* (DVII.US.5); “*Principalmente com carinho*” (DVII.US.5.1); *“Isto promove a cultura de paz”* (DVII.US.5.2).

Pela descrição anterior, compreende-se que o sujeito demonstra ter consciência de como hoje as pessoas carecem de contato com o outro, sobretudo com desvelo, e demonstra confiar na MS como estratégia para favorecer as interações humanas e como elemento propulsor ao desenvolvimento de uma cultura de paz.

Nessa direção, “[...] evidencia-se a educação como forma de conscientização, mobilização e prevenção em todos os níveis sociais, e as crianças como sujeitos que podem transportar a paz do patamar da imaginação para a realidade, ao serem cuidadas com amorosidade, o que pode favorecer desdobramentos posteriores”^(30)^.

Contudo, há identificação do participante, conforme mencionado na descrição e consideração sobre como a temática da MS é veiculada pela oficina, indicando o reconhecimento de seus benefícios para a coletividade: “*Achei extremamente interessante* [...] *e muito útil o tema proposto*” (DV.US.1).

Considerando sua dimensão teórica, no léxico teoria^(26)^ (do grego, *theoría*), substantivo feminino, diz respeito aos princípios básicos e elementares de uma arte ou ciência. Aqui, a MS, aliada a alguns princípios, qualifica o diálogo entre a teoria, a técnica e a prática das ações pedagógicas vivenciadas no programa MEB.

A teoria também pode ser compreendida como um sistema [...] que trata de princípios, considerando sistema^(26)^ (do grego, *sýstema*) como método, modo, forma ou plano e princípio^(26)^ (do latim, *principium*), aqui visto como fundamento, base da MS.

Esses princípios teóricos também se encontram imbricados com a técnica^(26)^ (substantivo feminino), ou seja, com a maneira ou habilidade especial de executar ou mesmo de ensinar a MS. Isso pode ser visualizada na descrição de outro participante da oficina: “*Gostei muito da técnica*” (DVI.US.1).

São princípios teórico-técnicos que, aplicados à MS, caracterizam também a didática empregada, ou seja, a ação prática como utilizada pelo programa MEB, a qual possibilita gradativamente o alcance dos efeitos desejados. Para sua compreensão, sugere-se que o profissional se sinta no lugar da mãe/bebê – estratégia didática utilizada ao apresentar a técnica –, ou seja, considere a maneira de ensinar os passos da MS de forma que a mesma seja realizada em conjunto, colocando todos como sujeitos da ação educativa.

Na perspectiva do profissional participante, a experiência na oficina trouxe descobertas: “[...] *uma técnica de terapia complementar que não conhecia a fundo*” (DV.US.2). Os participantes desvelam a MS como uma estratégia metodológica capaz de aproximar mãe e filho, contribuindo para a otimização do cuidado: “*Uma ferramenta para melhorar o vínculo mãe e filho e com isso melhorar as áreas de cuidado*” (DIX.US.2); “*Essa técnica simples que pode ajudar muito no contato com mãe e filho*” (DX.US.2).

Vislumbrando sua dimensão prá.ti.ca (do grego, *praktiké*, substantivo feminino), localiza-se no léxico^(26)^ a aplicação das regras ou dos princípios de uma arte ou ciência. Nessa mesma perspectiva, Leboyer, ao se reportar à MS, assim poetou:

A massagem dos bebês é uma arte

tão antiga quanto profunda.

Simples, mas difícil.

Difícil por ser simples.

Como tudo o que é profundo^(1)^

No léxico^(26)^, encontra-se também prá.ti.ca como habilidade [...] adquirida por prolongado exercício – nesse caso, compartilhada com os sujeitos participantes da oficina. Assim, “O saber prático de maneira diferente do saber teórico, ao não poder ser generalizado, poderá ser utilizado como diretriz e orientação^(22)^. Habilidade esta que se atinge de modo singular com o fazer, fazer, fazer...”

Em sua descrição, o participante da oficina sugere: “*E a parte prática muito boa também*” (DV.US.4). A utilização de bonecos para a aprendizagem foi considerada como adequada, o que encaminha o *fenômeno educação* a ser iluminado nesse espaçotempo da oficina.

Na sequência das descrições, nota-se que o participante destaca a prática da MS como “ação amorosa”, ocasião adequada para a troca de informações com o bebê por meio dos sentidos, bem como para observá-lo e percebê-lo: “*A massagem é um ato de amor*, *um momento de compartilhar experiências com o bebê, entendê-lo e respeitá-lo*. *Esta interação só traz benefícios e precisa ser valorizada e propagada*” (DVII.US.4). Desse modo, o participante da oficina demonstra estar ciente da reciprocidade e das vantagens que esta prática acarreta, ao enfatizar que a mesma deve ser estimulada, compartilhada e expandida.

Pode-se dizer, nesse contexto, que: “A prática, além de solicitar saberes prévios, que favoreçam o caráter teórico-técnico-prático, em dialogo, exige outros saberes que [...] servem como guia (ético)”^(22)^. A importância desses saberes foi identificada nas descrições: “[...] *muito rica as informações acerca do desenvolvimento sensorial, psicomotor que embasa os fundamentos da massagem*” (DI. US.3); “*E, mais ainda, mostra claramente os benefícios e fortalecimento do vínculo afetivo com o bebê*” (DI.US.4).

O profissional indica, em outra descrição, que, embora estivesse na expectativa de aprender apenas os passos da MS, constatou que os saberes foram fartos, com base na teoria disponibilizada sobre o DI: “[...] *me surpreendeu*, *esperava aprender somente a prática da massagem*” (DI.US.2). Tais saberes, acrescidos da compreensão das vantagens e da eficácia da intervenção para a ligação afetuosa da mãe com seu bebê, favorecem que o conhecimento avance qualitativamente de forma processual e dinâmica. Embora a MS seja um toque sistematizado sobre a pele, não se isolando dos demais sentidos, ela pode ser considerada uma *intervenção multimodal*, por favorecer a integração entre os sentidos e a motricidade^(17)^.

Em sintonia com as premissas da pedagogia sócio política freireana^(29)^, observa-se como o trabalho vem sendo desenvolvido de maneira comprometida, por meio de uma relação dialógica que focaliza a educação de forma igualitária e horizontal, expondo como os conhecimentos de natureza teórico-técnica-prática; quando integrados, possuem relevâncias entre si de igual teor.

Assim, ao refletir sobre o que muitas vezes ocorre na música, recorrendo a uma dimensão metafórica ou mesmo a uma variação imaginativa, pode-se vislumbrar quando as vozes ou os instrumentos musicais se alternam ou mesmo estabelecem um diálogo entre si. Como no jazz, é possível remeter à relação imbricada entre a teoria, a técnica e a prática da MS, o que nos possibilita também desvelar um diálogo: onde a teoria carrega o conhecimento milenar da massagem oriental, conjugada aos princípios elencados pelo programa MEB para sua realização. Já a técnica se caracterizou pela transmissão oral sistemática dos passos da MS, desde o oriente, de geração a geração, permitindo que a mesma chegasse até o ocidente e ainda continue a ser realizada e também ensinada até nossos dias. A prática se expressa na ação pedagógica por meio da MS, caracterizada por sua eficácia e sustentada pela teoria do DI, a partir da aplicação continuada da técnica no programa MEB, adaptada e compartilhada com famílias, estudantes e profissionais.

Entretanto, a escolha metodológica e a seleção dos referenciais teóricos trazidos para a temática em foco, sejam esses de caráter observacional, de aprendizado ou de descoberta, junto a fatores motivacionais, indicam a valorização dos sujeitos envolvidos nas ações da MS oferecidas pelas oficinas.

Dessa forma, pensar a ação educativa da MS nesse caminho exige uma preocupação com a educação inserida no contexto mais amplo de valorização das relações humanas, como destacado em outra descrição: “[...] *uma forma de valorizar a figura da mãe”* (DIX.US.3); e “*Melhorar vínculo com o profissional da saúde”* (DIX.US.4).

Como um elemento complementar, observa-se ainda outro profissional fazer referência à MS, não só para disponibilizar aos pais/mães-bebês, mas também como recurso para olhá-los e acolhe-los com atenção: “*Vai ser uma ferramenta a mais para* [observar] *e oferecer aos pacientes*” (DVI.US.4).

Em outros termos, isso significa que a compreensão de que sempre se sabe alguma coisa remete à possibilidade de saber a partir da experiência vivida e, portanto, poder usar esse conhecimento como ponto de partida para continuar, no processo^(29)^, aprendendo e, dessa maneira, manter a abertura de horizontes, o que possibilita identificar o que seria necessário para que o conhecimento avance^(22)^.

Tal ponto de vista solicita que a docente estude, contextualize, reflita, escreva, dialogue e investigue, pois sua função no ambiente pedagógico da extensão exige a busca de conhecimentos de diversas naturezas, com objetivos definidos, através de estratégias que conduzam à construção de conhecimentos originais^(11,17)^.

Todavia, observa-se a admiração do participante da oficina pelos fundamentos da MS, ao revelar como a experiência foi significativa em termos de ensinamentos e admitir que seria bom ter acesso a mais conhecimentos: “[...] *a parte teórica* [...] *foi maravilhosa e gostaria muito de saber mais*” (DV.US.3). Cabe, por fim, mencionar a descrição “*Ótima didática da palestrante*” (DVIII.US.1), que tece consideração acerca da atuação da professora com referência à estratégia pedagógica utilizada.

Nesse aspecto, o conhecimento é visto como produto da ação e reflexão diante de um problema (conflito cognitivo)^(29)^. Isso pode ser endossado pela compreensão dos participantes da oficina em relação à complexidade dos saberes que dão sustentação a essa ação educativa por meio da MS: “*O assunto abrange muitas coisas*” (DIV.US.2); “*Oficina rica em conhecimentos*” (DXI.US.1).

Nesse contexto, a oficina de MS se revela como um meio de aprendizagem com diversos propósitos: fazer com que os profissionais e estudantes compreendam que o paradigma dominante das ciências médicas, centrado em patologias, não responde a alguns de seus anseios sobre cuidados, e sobretudo, quais são as necessidades de saúde das famílias. Ela também enfatiza a importância do conhecimento de outras áreas do saber para que os participantes despertem para a dimensão humana em uma perspectiva interdisciplinar, ética e existencial. A oficina apresenta como se tem pensado e planejado intervenções de cuidado, para além da terapêutica clínica, com ações pautadas pelos conhecimentos do DI mediadas pela MS e situadas na perspectiva das competências do bebê, de seu *Ser*, considerado protagonista da ação. Dessa forma, valorizam-se suas expressões e capacidades de se relacionar precocemente e de ser parceiro, a fim de motivar a busca pela descoberta da criança pequena.

A compreensão do que pode acontecer com as famílias a partir do estabelecimento de um ritual diário de MS contribui para reforçar as atitudes positivas dos profissionais, despertando o interesse em enfrentar o desafio de construir um trabalho nessa direção. Isso é demonstrado pelo depoimento a seguir, em que a profissional afirma que a oficina despertou sua atenção, assegurando que irá (ela e a equipe) montar o GTMEB na UBS onde trabalha: “*Ótimo curso, muito interessante*” (DXIII.US.1): “*Vamos fazer com certeza na UBS*” (DXIII.US.2).

Procura-se, assim, cumprir a função social da academia, por meio da sensibilização de estudantes e profissionais que atuam em creches, escolas de educação infantil, unidades hospitalares e ambulatórios de UBS. Tais locais solicitam que se desenvolvam propostas pautadas pelo compromisso com a interdisciplinaridade como meio dos profissionais se constituírem em equipes.

No contexto do programa MEB, acredita-se que, ao tornar possível a articulação da pesquisa como ação educativa, no âmbito do ensino e da extensão, aliando conhecimentos empíricos e científicos sobre a temática da MS, pautada pelo DI, encontra-se a possibilidade de contribuição para a formação de profissionais da primeira infância, isto é, uma formação dirigida ao desenvolvimento humano^(22)^, com foco na otimização da parentalidade^(10)^.

Considerando a afetividade como necessidade fundamental de todos os seres humanos, indispensável à construção equilibrada da personalidade, pode-se destacar a alfabetização em termos da afetividade e ternura como um objetivo prioritário e um aspecto-chave de todo processo educacional. Além de sua influência no processo vital e de amadurecimento das pessoas, ela tem uma relação inequívoca com a convivência^(30)^.

Dessa maneira, ao considerar o vivido nas oficinas de massagem com bebês, evidencia-se como tal ação educativa pode se constituir um forte recurso auxiliar do profissional tanto no desenvolvimento da sua capacidade de estabelecer relações inteligentes entre os dados e informações obtidas na experiência quanto no âmbito dos conhecimentos construídos.

### Reflexões da Experiência Desvelada

A trajetória metodológica de pesquisa fenomenológica hermenêutica possibilitou iluminar as temáticas em suas variadas perspectivas, à procura da construção de intersignificações, bem como permitiu compreender a abordagem proposta à luz das demais formas de conceber os estudos científicos. Foi evidenciado um todo em que os significados puderam circular de acordo com os sentidos atribuídos a cada uma, desvelando o fenômeno da formação profissional humanizada com oficinas de MS: diálogos entre teoria, técnica e prática.

## CONCLUSÃO

Os sentidos dados às oficinas apontam perspectivas que auxiliam os profissionais de saúde, tanto no desenvolvimento da capacidade de estabelecer relações humanas nos espaços profissionais quanto na possibilidade de abertura ao compartilhamento das experiências vividas, acrescidas a outras que possam ser construídas em parcerias, situando o foco desta trajetória na dimensão sociopolítica dos vínculos afetivos, ainda hoje pouco compreendidos.

Nos cenários de formação do programa MEB, além de procurar despertar os graduandos e profissionais – participantes da oficina – para uma prática de cuidado mais humano, propõe-se desenvolver ações que organizem e integrem os novos conhecimentos aos já existentes, visando transformações. Daí emerge a possibilidade de que eles experienciem suas próprias ações como sujeitos, pois, ao se situarem e interagirem, constroem a si mesmos e fazem cultura. Eles percebem que o vínculo é também moral, contextual/ético e, inclusive, político.

## Data Availability

Todo o conjunto de dados que dá suporte aos resultados deste estudo está disponível mediante solicitação ao autor correspondente.

# Translation: Formación profesional humanizada con talleres de masaje Shantala: diálogos entre teoría, técnica y práctica

**DOI:** 10.1590/1980-220X-REEUSP-2025-0258es

**Authors:** Maria das Graças Barreto da Silva, Vitória Helena Cunha Espósito, Conceição Vieira da Silva Ohara

**Affiliations:** 1 Universidade Federal de São Paulo, Escola Paulista de Enfermagem, Departamento de Enfermagem Pediátrica, São Paulo, SP, Brasil.; 2 Pontifícia Universidade Católica de São Paulo, Faculdade de Educação, São Paulo, SP, Brasil.

**Keywords:** Shantala, Desarrollo Humano, Enfermería Pediátrica, Investigación Cualitativa, Educación

## Abstract

**Objetivo:**

Comprender las experiencias vividas por profesionales y estudiantes que participan en talleres de masaje infantil: una de las actividades del programa de extensión de Masaje y Estimulación con Bebés (MEB).

**Método:**

Enfoque fenomenológico hermenéutico basado en la pregunta: ¿Cómo fue tu experiencia en el taller de masaje infantil? A solicitud del Programa de Promoción de la Salud, se realizaron talleres de masaje Shantala en la ciudad de São José dos Campos, SP, con el objetivo de implementar la acción educativa del programa MEB, caracterizada por el Masaje Terapéutico y Grupos de Estimulación con Bebés en la rutina de atención maternoinfantil en Unidades Básicas de Salud.

**Resultados:**

Los significados otorgados a las experiencias vividas por los participantes de los talleres apuntan a perspectivas que les ayudan a desarrollar su capacidad para establecer relaciones humanas en espacios profesionales. Los participantes se construyen, se sitúan y crean cultura, reconociendo que el vínculo también es moral, contextual, ético y político.

**Consideraciones finales:**

En los escenarios de formación del programa MEB, se proponen diálogos entre teoría, técnica y práctica, organizando e integrando nuevos conocimientos con los existentes, con el objetivo de generar transformaciones.

## INTRODUCCIÓN

Como un saber culturalmente expresivo, que ha ido adquiriendo nuevos significados a lo largo de la historia, el masaje con bebés originario de la India, denominado masaje Shantala (MS)^(1)^, propició la construcción de un trabajo con enfoque corporal, realizado de manera sistematizada, por el programa de extensión universitaria Masaje y Estimulación con Bebés (MEB)^(2)^. Basado en los conocimientos sobre el desarrollo neuropsicomotor afectivo infantil, el programa MEB se compone de dos acciones educativas: el Grupo Terapéutico de Masaje y Estimulación con Bebés (GTMEB), que acoge a madres/padres con sus bebés lactantes; y los talleres de MS, con experiencias que introducen a profesionales y estudiantes de las áreas de salud y educación infantil en la temática de el MS.

Estos talleres, además de asesorar a profesionales y servicios institucionales, tienen un carácter itinerante. En el ámbito académico, como forma de preparar a los estudiantes para trabajar con las familias, el programa MEB se organiza en dos proyectos: “Grupo de Estudio de Masaje y Estimulación con Bebés”, de acción continua; y “MS: intervención multimodal y somatosensorialidad”, tema elegido anualmente por las monitoras para su trayectoria^(3)^.

Las experiencias vividas desde su origen, en 1996, todavía como proyecto de extensión^(4)^, continuaron como “programa MEB” a partir de 2012, con acciones educativas en ambulatorios orientados a la atención primaria en unidades hospitalarias pediátricas y neonatales, así como guarderías, además de otras actividades académicas desarrolladas con los cursos de licenciatura en los anfiteatros de la Escuela de Enfermería.

La literatura sobre el MS^(5)^ demuestra que la demanda de este tipo de intervención está ganando adeptos y que su uso terapéutico se difunde cada vez más, incluso en las Unidades de Terapia Intensiva Neonatal^(6,7)^. Sin embargo, estas unidades, a menudo, aún carecen de enfoques humanizados que consideren la salud como el fin último de los cuidados y que estén orientados al protagonismo de las familias. En esta perspectiva, también destaca cómo los profesionales tratan de expresar su insatisfacción con lo que suele ocurrir en las instituciones —de salud y educación infantil—, donde las expresiones de los bebés, así como las actitudes de los niños, no se utilizan como estrategias para cuidarlos.

Así, a medida que los profesionales de las áreas de salud y educación infantil, los estudiantes y las familias tomen conciencia de la calidad del contacto físico en los cuidados, de la importancia de la intervención mediada por el contacto físico y de sus efectos sobre el desarrollo infantil (DI), se fortalecerá el vínculo afectivo^(8,9)^. Sin embargo, con la ampliación y diversificación de las investigaciones centradas en estos temas, se espera que se reconozca la acción educativa, así como su valoración como tecnología ligera por sus beneficios, esenciales para el rol de cuidados que integran la parentalidad^(10)^.

En respuesta a estas inquietudes, se ha buscado, con los talleres de MS, hacer accesibles los conocimientos relacionados con el DI y, así, a través del MS^(11)^, evidenciar a los profesionales y estudiantes la necesidad de revisar las rutinas de cuidados y otros procedimientos utilizando los conocimientos de las franjas de edad infantiles a través de diálogos y reflexiones.

El Ministerio de Salud, con la publicación de la Ordenanza n.º 971, aprobó la Política Nacional de Prácticas Integrativas y Complementarias, con un enfoque de atención en el Sistema Único de Salud, que traza las directrices y responsabilidades para la implementación de sus acciones y servicios en el territorio nacional^(12)^. Cabe destacar que, en Brasil, existe desde hace tiempo, una creciente búsqueda por parte de la población de la legitimación del enfoque terapéutico holístico, incluida una demanda de Prácticas Integrativas y Complementarias en Salud (PICS)^(13)^. Con la publicación de la Ordenanza n.º 849, en 2017, el Ministerio de Salud amplió las PICS e incorporó el MS, contemplando a la población materno-infantil^(14)^. El marco legal representó un medio para concretar una estrategia de promoción del acceso, lo que supuso un gran logro para la salud pública.

Teniendo en cuenta la responsabilidad social de la academia en la formación de profesionales de la salud y la educación infantil para el cuidado humanizado^(15)^, se buscó contribuir a la mejora de los servicios como participantes en las políticas públicas orientadas a la primera infancia, uniendo esfuerzos para que la ley, que especifica la relevancia de la atención al DI, pueda expresarse en acciones^(16)^.

La difusión de los conocimientos producidos a través de las acciones desarrolladas por el programa MEB se ha presentado sistemáticamente a la comunidad científica, constituyendo como uno de sus resultados el trabajo “Los sentidos de las experiencias vividas en el programa de extensión de masaje y estimulación con bebés”^(17)^, del que se ha extraído el fragmento que aquí se presenta, titulado “Apoyo a la formación profesional humanizada: diálogos entre teoría, técnica y práctica”.

La motivación para realizar este estudio, con el objetivo de consolidar las actividades del programa MEB, permitió comprender el significado de las experiencias vividas en el taller de MS por quienes participaron en él.

## MÉTODO

### Diseño del Estudio

Cabe destacar, para esta investigación, la orientación fenomenológica, ya que los estudios académicos no deben limitarse única y prioritariamente a sus aspectos técnico-científicos, pues se considera que la enfermería, al igual que la educación, puede beneficiarse de la amplitud del cuidado orientado a la dimensión humana como ser situado existencialmente en el mundo. En este estudio, se optó, en particular, por la fenomenología existencial y hermenéutica, ya que dicha epistemología permite una mirada cualificada sobre una de las acciones educativas desarrolladas por el programa MEB. La fenomenología busca comprender a los seres humanos en su complejidad “[...] como seres situados en el mundo con los demás al admitir abordarlos en sus variadas perspectivas”^(18)^.

Esta trayectoria se encuentra respaldada por autores clásicos^(19)^, así como por pensadores contemporáneos^(18,20,21)^, cada uno con su propio hacer fenomenológico, con un rigor metodológico propio, característico de esta forma de investigar. Desde esta perspectiva, se destaca cómo el conocimiento se produce a partir de un lugar determinado, es decir, en el mundo tal y como se enfoca aquí, en situación^(22)^.

Con la introducción de la fenomenología en la hermenéutica, se observa el ser en la existencia, en la llamada ontología, es decir, el ser concebido como poseedor de una característica común, inherente a todos y cada uno de los seres^(19)^. De este modo, al hacer visible la estructura del ser en el mundo, se basa en la comprensión e interpretación de cómo se manifiestan las cosas^(18)^, lo que permite comprender el significado de las experiencias vividas en los talleres de masaje con bebés.

Al iluminar de manera comprensiva, este estudio hizo visible el trabajo desarrollado en el programa MEB, permitiendo comprender los significados de su construcción para quienes participan en él. Sistematizada en los espacios de los talleres, la acción educativa trajo la posibilidad de construir una trayectoria metodológica propia^(17)^ al enfatizar la praxis, es decir, la experiencia consciente del sujeto en su mundo-vida, recorrida con estudiantes y profesionales.

### Lugar, Población y Criterios de Inclusión

Esta trayectoria metodológica se refiere a los elementos responsables de la pertinencia y relevancia del texto en relación con el contexto en el que se produce, es decir, a su adecuación a la situación sociocomunicativa. Abarca cómo se accedió a las descripciones de los participantes, incluyendo, como región de investigación, el taller V. Este término se refiere al área temática, es decir, al conjunto de experiencias delimitadas que se están investigando mediante la metodología fenomenológica^(23)^.

A petición de la coordinación del Programa de Promoción de la Salud/Núcleo de Prácticas Integrativas y Complementarias/Departamento de Políticas de Salud de la Secretaría Municipal de Salud, se impartieron cinco talleres de MS en la ciudad de São José dos Campos, SP. Estos talleres, destinados a profesionales de la salud y estudiantes de enfermería, tenían como objetivo formar multiplicadores para operar con el MS integrada en los trabajos que ya desarrollaban con las embarazadas y en el segmento de la puericultura. De esta manera, se vislumbró la implementación del GTMEB en la rutina de cuidados de salud materno-infantil por parte de la red de atención primaria y secundaria, en las Unidades Básicas de Salud (UBS) y en la Unidad Casulo del Centro de Lactancia, donde ya trabajaban.

### Recolección de Datos

Las descripciones fenomenológicas fueron recopiladas por la investigadora, al final de cada evento, de los talleres I, II, III, IV y V, momento en el que los participantes respondían a la siguiente pregunta: ¿cómo fue su experiencia en el taller de MS?

Como el taller V fue el último en ofrecerse, de forma aleatoria, comenzamos la lectura de las descripciones de sus participantes, eligiéndolo como la región de investigación del estudio.

Dado que la investigación cualitativa no requiere un número determinado de participantes, el estudio pudo concluirse cuando los significados ya encontrados expresaron contenidos satisfactorios para la comprensión de sus estructuras esenciales^(24)^. Se procedió también a las lecturas iniciales de las descripciones de los participantes de los demás talleres, sin que se encontraran significados relevantes, es decir, que añadieran elementos a la comprensión del fenómeno. Por lo tanto, se puede considerar que una muestra cualitativa ideal es aquella que refleja, en cantidad e intensidad, las múltiples dimensiones de un fenómeno determinado, buscando la calidad de las acciones y las interacciones a lo largo de todo el proceso^(25)^ *.*

El criterio de inclusión de las descripciones de los 12 profesionales y una estudiante, participantes en el taller, contemplaba su participación en los dos módulos del taller V. De este modo, se siguió atentamente la trayectoria de sus descripciones, con la mirada puesta en sus puntos de vista. Para ello, se trató, sin juicios, de mantener en suspenso las opiniones, suposiciones e interpretaciones, centrándose en las experiencias vividas por ellos, es decir, en los significados relevantes que señalaban el fenómeno “[...] en respuesta a la pregunta de la investigación”^(17)^.

### Análisis y Tratamiento de los Datos

Desde esta perspectiva, la comprensión se produjo a través del sentido que surgió como resultado de la reducción fenomenológica, admitiéndose como un conjunto de afirmaciones significativas denominadas unidades de significado (US), que apuntan a la conciencia que el investigador tiene del fenómeno^(23)^. A continuación, con el apoyo del léxico^(26)^, se procuró que las US reflejaran ciertas dimensiones del contexto. Tras numerosas lecturas de las diferentes descripciones de los 13 participantes del taller V —identificadas como D (descripción) I a XIII (con números romanos que se refieren a los participantes del taller)—, se llegó a la primera reducción fenomenológica. De esta se derivaron de 1 a 59 US (presentadas por números cardinales) en respuesta a la pregunta de la investigación. Una vez organizadas, generaron síntesis, que están abiertas a la interpretación.

Tras proceder a una segunda reducción, se visualizaron 15 temas a partir de los elementos esenciales del fenómeno, expresados de la siguiente manera: teoría, técnica y práctica; aprendizaje; didáctica; estrategia metodológica; perspectiva interdisciplinaria; propósito; expectativa; descubrimiento; despertar; falta; felicitación; satisfacción; cuidado; evaluación; y agradecimiento*.* Debido a sus imbricación, al explicitar las convergencias, se buscó simultáneamente completar una textura a partir de estas temáticas, lo que redundó en la reorganización de grandes categorías que, a continuación, con la realización de una tercera reducción, apuntan a los temas, expresados de la siguiente manera: (1) Apoyo a la formación profesional humanizada: diálogos entre teoría, técnica y práctica; (2) Contribución a la construcción del vínculo madre/padre-hijo y cuidadores profesionales; y (3) Tiempo vivido en la experiencia del taller del MEB: desvelando significados. Sin embargo, para este trabajo, se presenta el primer tema expresado a continuación.

### Aspectos Éticos

El proyecto de este estudio fue aprobado por el Comité de Ética en Investigación de la Universidad Federal de São Paulo, bajo el dictamen n.º 1.424.084, y cumplió con los preceptos éticos de la Resolución n.º 466, de 12 de diciembre de 2012, del Consejo Nacional de Salud^(27)^.

## RESULTADOS Y DISCUSIÓN

Al contemplar el taller de MS como un espacio en busca de sentido, surgió lo que se había vivido expresado en el tema “Apoyo a la formación profesional humanizada: diálogos entre teoría, técnica y práctica”, a partir de los temas “teoría-técnica-práctica”, “aprendizaje”, “didáctica”, “estrategia metodológica” y “perspectiva interdisciplinaria”.

En esta trayectoria metodológica, al enfrentar el reto de la escritura, que se orienta pedagógicamente a la experiencia vivida, se compartió la siguiente idea: el acto de escribir reflexivamente sobre la práctica de vivir nos permite comprometernos con una praxis más reflexiva^(20)^. Por lo tanto, se considera que es a través de la escritura y la lectura que se puede mantener una relación conversacional; por lo tanto, esto está relacionado con la calidad del lenguaje y la escritura. Dicho de otra manera: está vinculado a la capacidad de la investigadora para dar sentido al tema en estudio (en tesis). De este modo, el lenguaje es el único medio por el cual podemos convertir la experiencia pedagógica en una forma simbólica que, a través de su naturaleza discursiva, nos permita crear una relación conversacional^(20)^.

Sin embargo, los conocimientos de la praxis relacionados con las ciencias del espíritu, en los que la ética es el elemento impulsor y guía de nuestro hacer^(28)^, al basarse en normas y reglas —es decir, en la moral—, pasan a orientar las diferentes formas de conducta, tanto de quien dirige como de quien realiza y recibe la acción práctica. De este modo:

El saber praxiológico de la phronesis, al estar basado en la excelencia de las acciones que logran encontrar los mejores “medios” para alcanzar un fin justo y ético, tiene como objetivo al hombre y sus experiencias, sin basarse en la exigencia de objetividad y de comprobación de lo que es, considerándolo como un ser de posibilidades, un ser situado en el tiempo y en el espacio, contextualizado, histórico, que vive en un mundo de incertidumbres^(28)^.

Sin embargo, en el saber ético de la práctica (phronesis) se encuentra la percepción de cómo se diferencia el concepto de cuidado en el espacio del taller. Allí, la dinámica se desarrolla entre los profesionales y la docente, utilizando muñecas para el aprendizaje práctico de la técnica de MS. Se observa que las instrucciones ofrecidas sirven de guía, ya que, al no ser determinantes, cuando se está en sesión de MS con las familias, pueden sufrir adaptaciones o modificaciones, según el contexto y las circunstancias manifestadas por los participantes. Con ello, se llama la atención para que los participantes observen las reacciones y expresiones de la madre/padre en relación con los toques, para que se respeten sus señales de placer y disgusto. Del mismo modo, se observan las de los bebés, teniendo en cuenta sus preferencias, ya que esto favorece el perfeccionamiento de la acción práctica, así como la del estudiante/profesional del cuidado, ayudándole a tomar las mejores decisiones.

Además, ante los trabajos desarrollados en el programa MEB, se destaca la importancia de que cada miembro del equipo se reconozca en el desarrollo de las acciones de cuidado como poseedor y productor de conocimientos significativos, estableciendo intercambios a través de diálogos sistemáticos, en un proceso en el que el intercambio de experiencias es primordial^(29)^.

Este ambiente propicio para el intercambio de experiencias y conceptos permite reinterpretar los significados dados a lo vivido, haciendo viable el respeto por la espontaneidad de los participantes en el taller y resaltando la comunicación entre las acciones ciudadanas y profesionales.

Dado que los participantes encuestados son profesionales y estudiantes con bagaje de conocimientos, ante nueva información que presenta sentido —aquí tomado en el sentido de “conciencia de”^(22)^—, realizan un esfuerzo por asimilarla. Así, ante un problema, indican la necesidad de superarlo, como se expone: “*Al ser humano moderno le falta el contacto*” (DVII.US.5); “*Sobre todo con cariño*” (DVII.US.5.1); “*Esto promueve la cultura de la paz*” (DVII.US.5.2).

Por la descripción anterior, se comprende que el sujeto demuestra ser consciente de cómo hoy en día las personas carecen de contacto con los demás, sobre todo con cariño, y demuestra confiar en la MS como estrategia para favorecer las interacciones humanas y como elemento impulsor del desarrollo de una cultura de paz.

En este sentido, “[...] se evidencia la educación como forma de concientización, movilización y prevención en todos los niveles sociales, y a los niños como sujetos que pueden transportar la paz del plano de la imaginación a la realidad, al ser cuidados con amor, lo que puede favorecer desarrollos posteriores”^(30)^.

Sin embargo, se identifica al participante, tal y como se menciona en la descripción y consideración sobre cómo se transmite el tema de la MS en el taller, lo que indica el reconocimiento de sus beneficios para la colectividad: “*Me pareció extremadamente interesante* [...] *y muy útil el tema propuesto*” (DV.US.1).

Considerando su dimensión teórica, en el léxico, la teoría^(26)^ (del griego, *theoría*), sustantivo femenino, se refiere a los principios básicos y elementales de un arte o ciencia. Aquí, el MS, junto con algunos principios, califica el diálogo entre la teoría, la técnica y la práctica de las acciones pedagógicas vividas en el programa MEB.

La teoría también puede entenderse como un sistema [...] que trata de principios, considerando sistema^(26)^ (del griego, *sýstema*) como método, modo, forma o plan y principio^(26)^ (del latín, *principium*), aquí visto como fundamento, base del MS.

Estos principios teóricos también se entrelazan con la técnica^(26)^ (sustantivo femenino), es decir, con la manera o habilidad especial de ejecutar o incluso de enseñar el MS. Esto se puede visualizar en la descripción de otro participante del taller: “*Me gustó mucho la técnica*” (DVI.US.1).

Son principios teórico-técnicos que, aplicados a el MS, también caracterizan la didáctica empleada, es decir, la acción práctica tal y como la utiliza el programa MEB, que permite alcanzar gradualmente los efectos deseados. Para su comprensión, se sugiere que el profesional se ponga en el lugar de la madre/bebé —estrategia didáctica utilizada al presentar la técnica—, es decir, que considere la forma de enseñar los pasos del MS de manera que se realice de forma conjunta, situando a todos como sujetos de la acción educativa.

Desde la perspectiva del profesional participante, la experiencia en el taller trajo consigo descubrimientos: “[...] *una técnica de terapia complementaria que no conocía en profundidad*” (DV.US.2). Los participantes revelan el MS como una estrategia metodológica capaz de acercar a la madre y al hijo, contribuyendo a la optimización del cuidado: “*Una herramienta para mejorar el vínculo entre madre e hijo y, con ello, mejorar las áreas de cuidado*” (DIX.US.2); “*Esta técnica sencilla que puede ayudar mucho en el contacto entre madre e hijo*” (DX.US.2).

Vislumbrando su dimensión práctica (del griego *praktiké*, sustantivo femenino), se encuentra en el léxico^(26)^ la aplicación de las reglas o principios de un arte o ciencia. En esta misma perspectiva, Leboyer, al referirse al masaje infantil, escribió:

El masaje infantil es un arte

tan antiguo como profundo.

Sencillo, pero difícil.

Difícil por ser simple.

Como todo lo que es profundo^(1)^

En el léxico^(26)^, también se encuentra prá.ti.ca como habilidad [...] adquirida por ejercicio prolongado, en este caso, compartida con los participantes del taller. Así, “El saber práctico, a diferencia del saber teórico, al no poder generalizarse, puede utilizarse como directriz y orientación^(22)^.

Habilidad que se alcanza de manera singular con el hacer, hacer, hacer...”

En su descripción, el participante del taller sugiere: “*Y la parte práctica también muy buena*” (DV.US.4). El uso de muñecos para el aprendizaje se consideró adecuado, lo que lleva al *fenómeno de la educación* a ser iluminado en este espacio-tiempo del taller.

A continuación de las descripciones, se observa que el participante destaca la práctica del masaje como una “acción amorosa”, una ocasión adecuada para intercambiar información con el bebé a través de los sentidos, así como para observarlo y percibirlo: “*El masaje es un acto de amor*, *un momento para compartir experiencias con el bebé, comprenderlo y respetarlo*. *Esta interacción solo aporta beneficios y debe valorarse y difundirse*” (DVII.US.4). De este modo, el participante del taller demuestra ser consciente de la reciprocidad y las ventajas que conlleva esta práctica, al enfatizar que debe estimularse, compartirse y expandirse.

En este contexto, se puede decir que: “La práctica, además de requerir conocimientos previos que favorezcan el carácter teórico-técnico-práctico, en diálogo, exige otros conocimientos que [...] sirven de guía (ético)”^(22)^. La importancia de estos conocimientos se identificó en las descripciones: “[...] *muy rica la información sobre el desarrollo sensorial y psicomotor que sustenta los fundamentos del masaje*” (DI.US.3); “*Más aún, muestra claramente los beneficios y el fortalecimiento del vínculo afectivo con el bebé*” (DI.US.4).

El profesional indica, en otra descripción, que, aunque esperaba aprender solo los pasos del MS, constató que los conocimientos eran abundantes, basados en la teoría disponible sobre el DI: “[...] *me sorprendió*, *esperaba aprender solo la práctica del masaje*” (DI.US.2). Estos conocimientos, sumados a la comprensión de las ventajas y la eficacia de la intervención para el vínculo afectivo de la madre con su bebé, favorecen que el conocimiento avance cualitativamente de forma procesual y dinámica. Aunque el MS es un toque sistematizado sobre la piel, sin aislarse de los demás sentidos, puede considerarse una *intervención multimodal*, ya que favorece la integración entre los sentidos y la motricidad^(17)^.

En sintonía con las premisas de la pedagogía sociopolítica freireana^(29)^, se observa cómo el trabajo se ha desarrollado de manera comprometida, a través de una relación dialógica que se centra en la educación de forma igualitaria y horizontal, exponiendo cómo los conocimientos de naturaleza teórico-técnico-práctica, cuando se integran, tienen relevancias entre sí de igual contenido.

Así, al reflexionar sobre lo que a menudo ocurre en la música, recurriendo a una dimensión metafórica o incluso a una variación imaginativa, se puede vislumbrar cuando las voces o los instrumentos musicales se alternan o incluso establecen un diálogo entre sí. Al igual que en el jazz, es posible remitirse a la relación entrelazada entre la teoría, la técnica y la práctica del MS, lo que también nos permite desvelar un diálogo: donde la teoría conlleva el conocimiento milenario del masaje oriental, combinado con los principios enumerados por el programa MEB para su realización. La técnica, por su parte, se caracterizó por la transmisión oral sistemática de los pasos del MS, desde Oriente, de generación en generación, lo que le ha permitido llegar a Occidente y seguir realizándose y enseñándose hasta nuestros días. La práctica se expresa en la acción pedagógica a través de la MS, caracterizada por su eficacia y sustentada por la teoría del DI, a partir de la aplicación continua de la técnica en el programa MEB, adaptada y compartida con familias, estudiantes y profesionales.

Sin embargo, la elección metodológica y la selección de los referentes teóricos aportados al tema en cuestión, ya sean de carácter observacional, de aprendizaje o de descubrimiento, junto con factores motivacionales, indican la valoración de los sujetos involucrados en las acciones de la MS ofrecidas por los talleres.

De este modo, pensar la acción educativa de la MS en este sentido exige una preocupación por la educación insertada en el contexto más amplio de la valoración de las relaciones humanas, como se destaca en otra descripción: “[...] *una forma de valorar la figura de la madre”* (DIX.US.3); y “*Mejorar el vínculo con el profesional de la salud”* (DIX.US.4).

Como elemento complementario, se observa que otro profesional hace referencia a la MS, no solo para ponerla a disposición de los padres/madres y bebés, sino también como recurso para observarlos y acogerlos con atención: “*Será una herramienta más para* [observar] *y ofrecer a los pacientes*” (DVI.US.4).

En otros términos, esto significa que la comprensión de que siempre se sabe algo remite a la posibilidad de saber a partir de la experiencia vivida y, por lo tanto, poder utilizar ese conocimiento como punto de partida para continuar, en el proceso^(29)^, aprendiendo y, de esa manera, mantener la apertura de horizontes, lo que permite identificar lo que sería necesario para que el conocimiento avance^(22)^.

Este punto de vista requiere que el docente estudie, contextualice, reflexione, escriba, dialogue e investigue, ya que su función en el entorno pedagógico de la extensión exige la búsqueda de conocimientos de diversa índole, con objetivos definidos, a través de estrategias que conduzcan a la construcción de conocimientos originales^(11,17)^.

Sin embargo, se observa la admiración del participante del taller por los fundamentos del MS, al revelar lo significativa que fue la experiencia en términos de enseñanzas y admitir que sería bueno tener acceso a más conocimientos: “[...] *la parte teórica* [...] *fue maravillosa y me gustaría mucho saber más*” (DV.US.3). Por último, cabe mencionar la descripción “*Excelente didáctica de la ponente*” (DVIII.US.1), que valora la actuación de la profesora en referencia a la estrategia pedagógica utilizada.

En este aspecto, el conocimiento se considera producto de la acción y la reflexión ante un problema (conflicto cognitivo)^(29)^. Esto puede respaldarse con la comprensión de los participantes del taller en relación con la complejidad de los conocimientos que sustentan esta acción educativa a través del MS: “*El tema abarca muchas cosas*” (DIV.US.2); “*Taller rico en conocimientos*” (DXI.US.1).

En este contexto, el taller de MS se revela como un medio de aprendizaje con diversos propósitos: hacer que los profesionales y los estudiantes comprendan que el paradigma dominante de las ciencias médicas, centrado en las patologías, no responde a algunas de sus inquietudes sobre los cuidados y, sobre todo, cuáles son las necesidades de salud de las familias. También enfatiza la importancia del conocimiento de otras áreas del saber para que los participantes tomen conciencia de la dimensión humana desde una perspectiva interdisciplinaria, ética y existencial. El taller presenta cómo se han pensado y planificado las intervenciones de cuidado, más allá de la terapéutica clínica, con acciones basadas en los conocimientos de la DI mediados por el MS y situados en la perspectiva de las competencias del bebé, de su *Ser*, considerado protagonista de la acción. De esta manera, se valoran sus expresiones y capacidades para relacionarse precozmente y ser un compañero, con el fin de motivar la búsqueda del descubrimiento del niño pequeño.

La comprensión de lo que puede suceder con las familias a partir del establecimiento de un ritual diario de MS contribuye a reforzar las actitudes positivas de los profesionales, despertando el interés por afrontar el reto de construir un trabajo en esta dirección. Esto queda demostrado en el siguiente testimonio, en el que la profesional afirma que el taller despertó su atención, asegurando que ella y su equipo montarán el GTMEB en el UBS donde trabaja: “*Excelente curso, muy interesante*” (DXIII.US.1): “*Lo haremos sin duda en la UBS*” (DXIII.US.2).

De este modo, se busca cumplir la función social de la academia, mediante la sensibilización de estudiantes y profesionales que trabajan en guarderías, escuelas de educación infantil, unidades hospitalarias y ambulatorios de UBS. Estos lugares solicitan que se desarrollen propuestas basadas en el compromiso con la interdisciplinariedad como medio para que los profesionales se constituyan en equipos.

En el contexto del programa MEB, se cree que, al hacer posible la articulación de la investigación como acción educativa, en el ámbito de la enseñanza y la extensión, combinando conocimientos empíricos y científicos sobre el tema de la MS, guiados por el DI, existe la posibilidad de contribuir a la formación de profesionales de la primera infancia, es decir, una formación dirigida al desarrollo humano^(22)^, con enfoque en la optimización de la parentalidad^(10)^.

Considerando la afectividad como una necesidad fundamental de todos los seres humanos, indispensable para la construcción equilibrada de la personalidad, se puede destacar la alfabetización en términos de afectividad y ternura como un objetivo prioritario y un aspecto clave de todo proceso educativo. Además de su influencia en el proceso vital y de maduración de las personas, tiene una relación inequívoca con la convivencia^(30)^.

De esta manera, al considerar lo vivido en los talleres de masaje con bebés, se evidencia cómo esta acción educativa puede constituir un fuerte recurso auxiliar para el profesional, tanto en el desarrollo de su capacidad para establecer relaciones inteligentes entre los datos y la información obtenidos en la experiencia, como en el ámbito de los conocimientos construidos.

### Reflexiones Sobre la Experiencia Revelada

La trayectoria metodológica de la investigación fenomenológica hermenéutica permitió iluminar los temas en sus diversas perspectivas, en busca de la construcción de intersignificaciones, y permitió comprender el enfoque propuesto a la luz de otras formas de concebir los estudios científicos. Se evidenció un todo en el que los significados pudieron circular de acuerdo con los sentidos atribuidos a cada uno, desvelando el fenómeno de la formación profesional humanizada con talleres de MS: diálogos entre teoría, técnica y práctica.

## CONCLUSIÓN

Los significados atribuidos a los talleres apuntan a perspectivas que ayudan a los profesionales de la salud, tanto en el desarrollo de la capacidad de establecer relaciones humanas en los espacios profesionales como en la posibilidad de abrirse al intercambio de experiencias vividas, sumadas a otras que puedan construirse en colaboración, situando el foco de esta trayectoria en la dimensión sociopolítica de los vínculos afectivos, aún hoy poco comprendidos.

En los escenarios de formación del programa MEB, además de tratar de despertar a los estudiantes y profesionales —participantes en el taller— hacia una práctica de cuidado más humana, se propone desarrollar acciones que organicen e integren los nuevos conocimientos a los ya existentes, con miras a la transformación. De ahí surge la posibilidad de que experimenten sus propias acciones como sujetos, ya que, al situarse e interactuar, se construyen a sí mismos y crean cultura. Se dan cuenta de que el vínculo es también moral, contextual/ético e, incluso, político.

## Data Availability

El conjunto de datos que respaldan las conclusiones de este estudio está disponible previa solicitud al autor correspondiente.
